# Light People: Prof. Kei May Lau, newly elected US NAE member in Hong Kong, talks about future of photonics and women in science

**DOI:** 10.1038/s41377-024-01401-9

**Published:** 2024-03-11

**Authors:** Yating Wan, Chenzi Guo

**Affiliations:** 1 Electrical and Computer Engineering, the Computer, Electrical and Mathematical Sciences and Engineering Division, King Abdullah University of Science and Technology, 23955-6900 Thuwal, Saudi Arabia; 2 Changchun Institute of Optics, Fine Mechanics and Physics, Chinese Academy of Sciences, Changchun, China

**Keywords:** Silicon photonics, Quantum dots, Semiconductor lasers

## Abstract

Photonics technology remains a driving force in today’s scientific landscape, marked by continuous innovation and cross-disciplinary relevance. In an enlighting conversation with Light: Science & Applications, Prof. Kei May Lau, a pioneer in photonics research, shares her deep insights on the evolution of technologies of LEDs, lasers, challenges of hetero-epitaxy, and the future of micro-LEDs and quantum dot lasers. Recently honored as a member of the US National Academy of Engineering (NAE) for her significant contributions to photonics and electronics using III-V semiconductors on silicon, Prof. Lau stands out as the sole Hong Kong scholar inducted into the NAE this year, joining 114 new and 21 international members. In this exclusive Light People interview, Prof. Lau shares her journey as a pioneering woman in engineering, her commitment to mentorship and academia, and her perspective on advancing female representation in science. The summary provided is distilled from Prof. Lau’s thoughtful responses during the interview. For a deeper exploration of Prof. Lau’s experiences and advice, the full interview is available in the Supplementary material.

**Figure:**
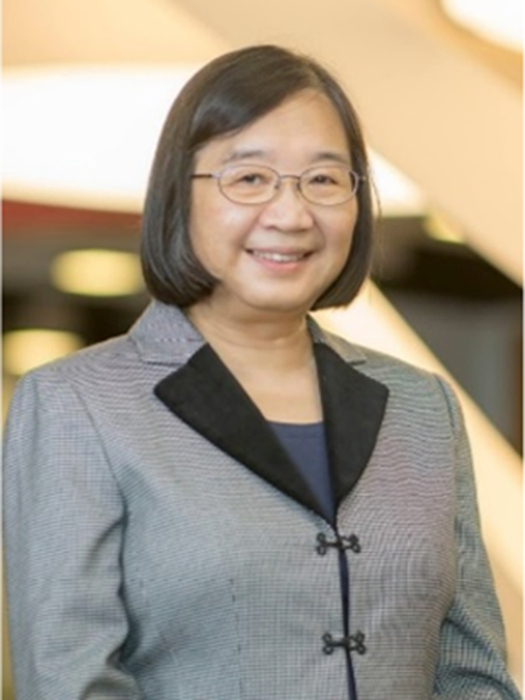

**Short Bio:** Prof. Kei May Lau is a Research Professor at the Hong Kong University of Science & Technology (HKUST). She received her degrees from the University of Minnesota and Rice University and served as a faculty member at the University of Massachusetts/Amherst until 2000. Prof. Lau is a Fellow of the IEEE, Optica (formerly OSA), and the Hong Kong Academy of Engineering Sciences. She is also a recipient of the IPRM award, IET J J Thomson medal for Electronics, Optica (formerly OSA) Nick Holonyak Jr. Award, IEEE Photonics Society Aron Kressel Award, US National Science Foundation (NSF) Faculty Awards for Women (FAW) Scientists and Engineers, and Hong Kong Croucher Senior Research Fellowship. She was an Editor of the IEEE Transactions on Electron Devices (1996–2002) and Electron Device Letters (2016–2019), an Associate Editor for the Journal of Crystal Growth and Applied Physics Letters. Prof. Lau’s research work is focused on the development of monolithic integration of semiconductor quantum devices on industry-standard silicon substrates.

**Q1: As a world-renowned expert in hetero-epitaxy of III–V on silicon by metal-organic chemical vapor deposition (MOCVD) for photonic devices, can you briefly introduce your research?**

**A1:** Sure, my research focus is on the hetero-epitaxial growth of III–V semiconductors on silicon using MOCVD, with an emphasis on photonic devices. I’ve dedicated many years to mastering epitaxy for device applications, and the integration of III–V materials on silicon is a key part of that. It’s a complex challenge, but we’re achieving increasingly positive outcomes. Our recent focus on the Lateral Aspect Ratio Trapping (LART) technique that allows selective growth of III–V devices laterally on top of the oxide layer is particularly interesting.

**Q2: You are one of the early contributors of MOCVD technology, essential in producing LEDs, high-speed transistors, and high-performance lasers. What inspired you to pursue this field?**

**A2:** My foray into MOCVD began during my PhD, when my advisor visited Cornell for his sabbatical and took me along. It was there I met Prof. Lester Eastman, my advisor’s mentor. They emphasized the importance of material control in semiconductor devices research, from design to growth. Faced with the choice between MBE, which was commercially available but expensive, and MOCVD, which allowed for custom-built systems, I opted for the more economical route with MOCVD – building my own system with top-of-the-line components. That decision kickstarted my journey in this field.

**Q3: Your early work includes pioneering research in LED technology, contributing to solid-state lighting and the development of components for high-speed computers and mobile units. Many micro-LED startups have been founded by your students. Could you share some insights into this?**

**A3:** My journey into LEDs began after working with high-frequency and other III–V devices, including lasers. An insightful ‘90 s article by Jeffrey Tsao and Roland Haitz discussed LED potential in lighting—deemed an unrealistic idea at the time. Yet, I saw its feasibility. This led me into LED research, and as the industry evolved, producing LEDs of increasing power and brightness, I recognized that academia was lagging behind. I decided to explore the opposite direction with micro-LEDs, aiming for high-brightness micro-displays. We started our micro-LED micro-display research almost two decades ago. It caught on, and we continued to refine the technology, achieving higher resolution and transitioning from monochromatic to full color displays.

**Q4: Your recent work also involves monolithic integration using QD materials on silicon. Can you outline the key challenges in this area, the global issues that capture your interest, and your plans for future research?**

**A4:** I think the challenge for quantum dot (QD) lasers, especially when using MOCVD, is achieving consistent reproducibility, which is likely why they haven’t been commercialized like quantum well lasers. Addressing this is one of the quantum dot laser’s broad challenges. Currently, my team is exploring lateral aspect ratio trapping to obtain defect-free III–V layers on buried oxide, which could significantly benefit lasers, detectors, and other components for Silicon photonic integration. It’s a thrilling avenue of research that we’re pursuing with great interest.

**Figure:**
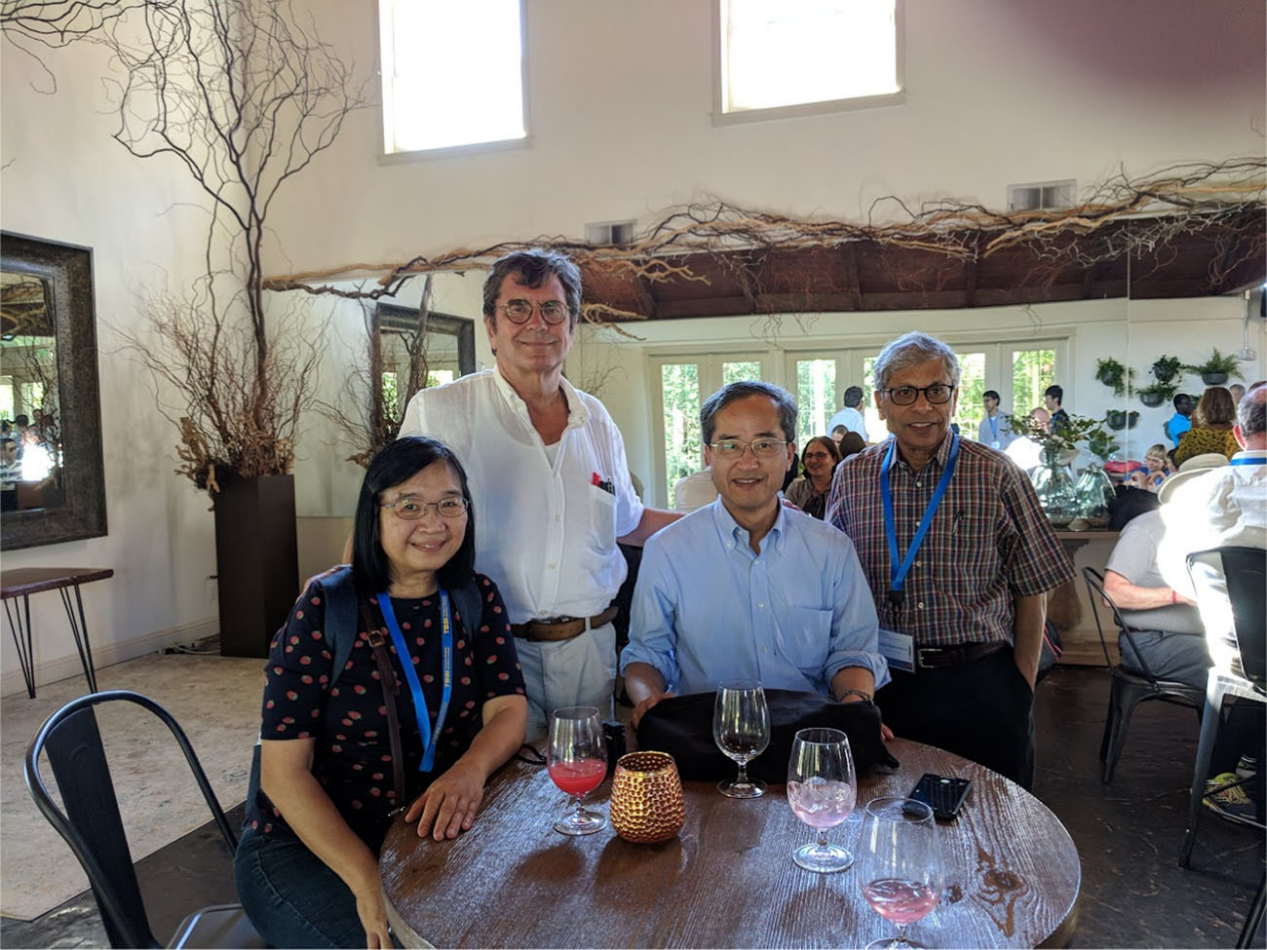
Prof. Kei May Lau, Prof. Dieter Bimberg, Prof. Yasuhiko Arakawa, and Prof. Pallab Bhattacharya

**Q5: Where did your initial interest in science/technology come from?**

**A5:** To be honest, my interest was quite broad during high school; I hadn’t pinpointed my favorite subject. It wasn’t until I attended the University of Minnesota that I leaned towards science and engineering, which I found straightforward because it primarily involved math and calculations – the common language for scientists and engineers. Physics became my major after a few switches, primarily because it allowed me to graduate a bit over three years, alleviating the financial burden of tuition for my parents. That practicality was my main motivation at the time.

**Q6: Considering that industry positions often offer triple the pay compared to academia, what influenced your decision to pursue a career in academia over industry?**

**A6**: After graduating, I spent two years in the industry. It was an enlightening period; I learned about product development and worked on government-funded projects. However, the primary focus on product delivery eventually became a source of frustration for me. I realized that in academia, I wouldn’t be bound by product timelines and could dedicate myself to my projects. That freedom to explore research interests without the constraints of commercial objectives is what drew me to a university career.

**Q7: Is there any particular challenge in your research career that is most memorable to you?**

**A7:** In research, challenges are a constant. I have a sign on my office door that reads, “All roads are winding,” which is a reminder that challenges persist throughout life. The most memorable aspect for me is learning to navigate these challenges. It’s crucial to recognize when you’re at a crossroad and to make informed decisions about when to pivot and which new direction to take.

**Q8: You spent your first sabbatical leave in 1989 at the MIT Lincoln Laboratory. How did this experience influence your subsequent scientific career?**

**A8:** I went there for the first sabbatical, that experience was great. I joined a group specializing in lasers and related devices and gained hands-on experience in processing. The generosity and support of the people were phenomenal; they shared their expertise, lent me equipment like masks, and taught me laser testing techniques. They had a saying before testing: “Let there be light,” and indeed, there was light. That camaraderie and learning experience with such a wonderful group had a lasting influence on my career.

**Q9: You joined HKUST in 2000 after years of industrial and academic experience in Massachusetts and founded the Photonics Technology Center. Why did you later consider to HKUST?**

**A9:** Returning to Hong Kong, where I was born and raised, felt like a homecoming. During a six-month visit, I was impressed by HKUST’s excellent facilities, which I believed would enable me to achieve more in my research. In Massachusetts, I often worked in isolation on device-related projects, but at HKUST, I saw an opportunity for greater collaboration and productivity. Additionally, family played a crucial role in this decision—raising my daughter to be bilingual and bicultural in the U.S. was challenging, and relocating to Hong Kong provided us with support like home-stay nannies, easing the burdens of daily chores and childcare.

**Figure:**
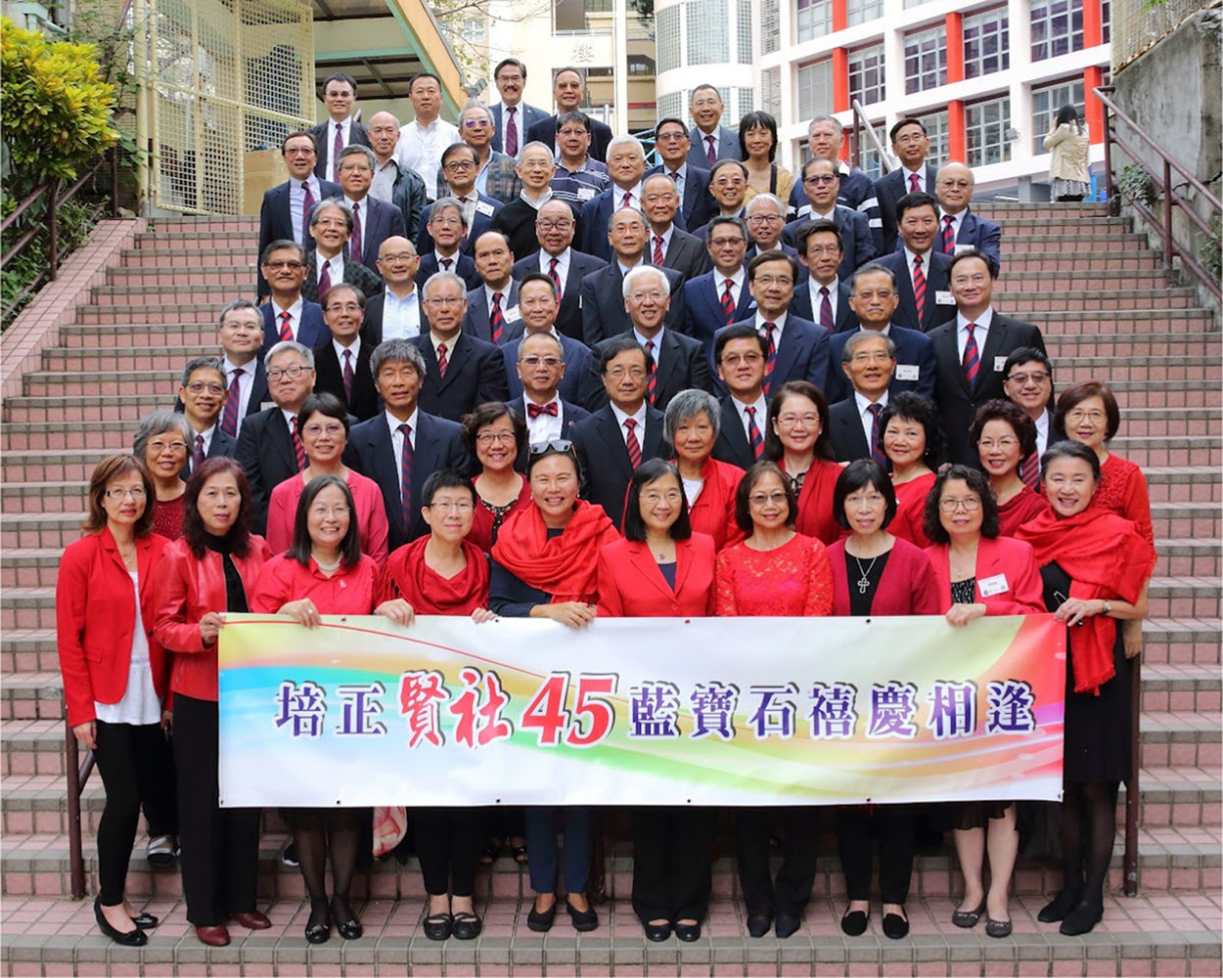
Prof. Lau participated in her high school reunion in Hong Kong

**Q10: After moving to HKUST, your career became more and more successful. You have received many awards, to name a few, the IEEE Photonics Society Aron Kressel Award (2017), and OSA’s Nick Holonyak, Jr. Award (2020). What career achievement are you most proud of?**

**A10:** I am most proud of the legacy of my students and postdocs. The real triumph, for me, is witnessing the transformation of the individuals who have passed through my lab. They arrive with potential and leave making substantial impacts. Seeing them flourish professionally and contribute meaningfully to our field is, without a doubt, my greatest accomplishment.

**Q11: Many universities in Hong Kong are starting transition to the Great Bay Area, do you have any suggestions for the promotion of collaborations?**

**A11:** Effective communication is crucial, particularly among the right individuals. Faculty members are independent thinkers, they might need assistance in identifying partners who share mutual interests. Once these connections are made, they are more likely to successfully collaborate and innovate together.

**Q12: In your opinion, how to promote the Industry-University-Research?**

**A12:** Communication is key. It’s essential that industry professionals, university scholars, and researchers develop a mutual understanding. Projects should align with the interests and needs of all three parties to ensure productive collaboration and meaningful outcomes.

**Q13: It’s enlightening to hear about your research perspectives. With both you and your husband being esteemed professors and leading busy professional lives, how do you maintain a work-life balance?**

**A13:** The flexibility offered by academia is a significant advantage in balancing our lives. We prioritize tasks as they come. Many people asked how I managed time, but actually I don’t really manage anything, including time. Whatever needs to be done, I just get it done. If I don’t get it done, okay, I don’t get it done, so be it.

**Q14: Facing the pressures of an academic career can be challenging. How do you cope with stress, and what hobbies do you enjoy outside of your research?**

**A14:** Maybe it’s my personality, I accept pressure as a part of the job and simply do my best. If tasks remain incomplete, I’m at peace with it, knowing I’ve done what I could. Outside of research, I enjoy traveling and socializing with friends. I also cherish the time spent with my daughter, supporting her in her activities. I don’t particularly gravitate towards sports or music, but I find relaxation in these simple pleasures.

**Q15: Regarding your daughter, have you ever encouraged her to pursue a career in engineering or research?**

**A15:** Not really, I believe children may not always want to follow their parents’ advice. If she seeks my input, I’m happy to share my thoughts, but I’ve never directed her toward a specific path. Instead, I’ve outlined the options and what those paths could lead to. She chose engineering on her own, and as for the future, her interests may evolve, and that’s perfectly fine.

**Figure:**
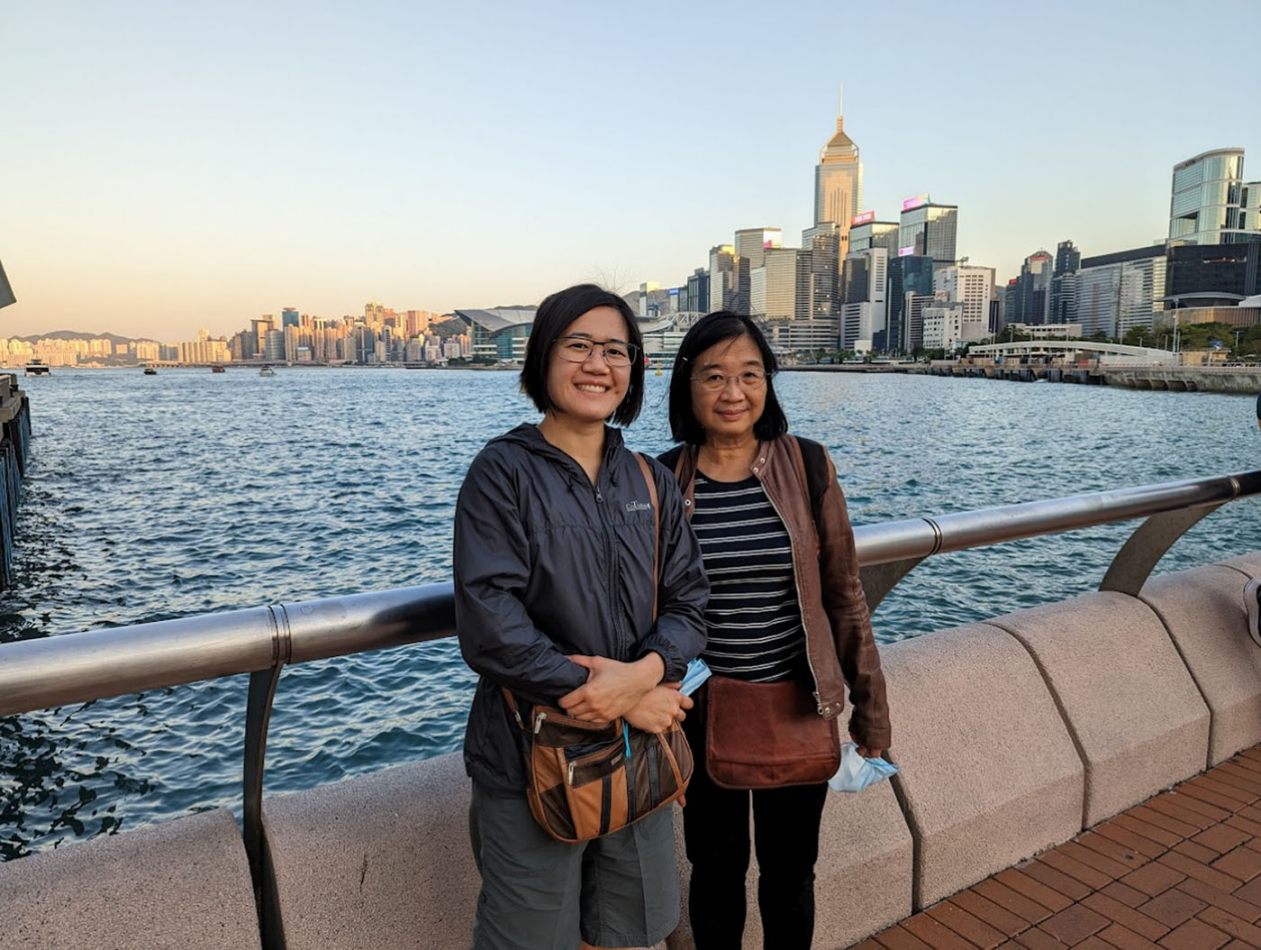
Prof. Lau and her daughter

**Q16: As a mentor, how do you tailor your guidance to accommodate the unique personalities and needs of different students?**

**Q16:** I take inspiration from Confucius’ principle, “Yin Cai Si Jiao,” which means to teach students in accordance with their aptitudes. Some students need more pushing and motivation while others don’t. So you have to motivate and challenge some students, or brainstorm with some other students without pushing too much. That’s how I interact with the students.

**Figure:**
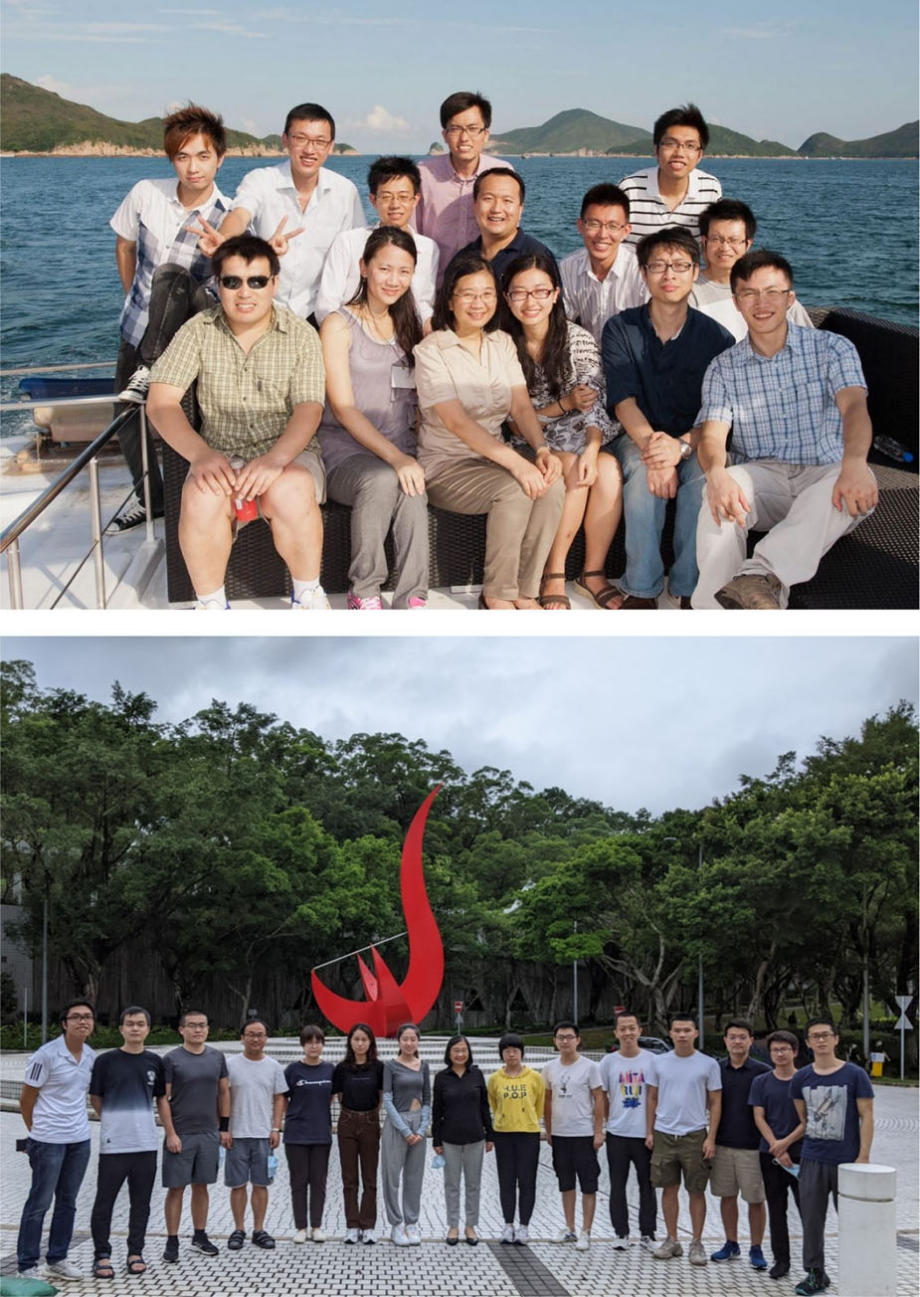
Prof. Lau and her students

**Q17: What qualities do you expect from the students when they graduate?**

**A17:** I expect them to be adept in their training, particularly with experimental work. They should be able to leverage this training to think critically and solve problems effectively. Employers value graduates not necessarily for their thesis topics, which may not directly align with a job, but for their proven ability to tackle challenges and find solutions. That’s the hallmark of a good education and what I hope for all my students.

**Q18: What are the criteria when you hire a student?**

**A18:** Motivation is a key criterion for me, provided their academic performance is above a certain threshold. I look for students who are genuinely eager to learn, not just those looking to spend a few years in school. When prospective PhD candidates ask about the duration of their studies, my response is that it varies—I’ve seen it completed in just over three years, while others may take seven or eight. Pursuing a PhD is a commitment to learning, not a sentence. So rather than asking how long it will take, I encourage them to focus on what they can achieve during their time in the program.

**Q19: What suggestions would you like to give for young professionals?**

**A19:** My advice is to actively seek out what truly interests you and immerse yourself in it. There’s wisdom in the lyrics of a song from ‘Mary Poppins’ I remember from childhood: “I do what I like, and I like what I do.” It’s a simple yet profound principle. Granted, it’s not always possible to align every aspect of your job with your passions, but striving to find joy in your work is essential.

**Q20: You are featured in a lot of local magazines in HK, as one of the Most Successful Women in Hong Kong and Greater China and have played a leading role in driving positive changes to the society. But in your early days, who is your role model?**

**A20:** Professor Evelyn Hu has been a profound role model for me. Her kindness, intelligence, and generosity toward younger and junior individuals in the field have always inspired me.

**Figure:**
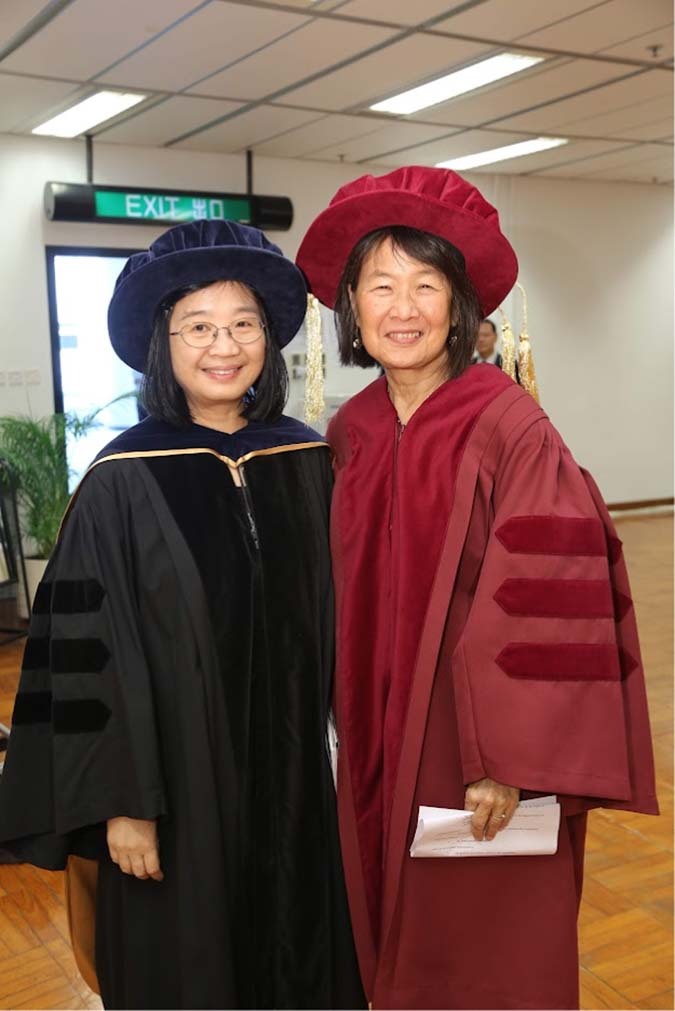
Prof. Kei May Lau and Prof. Evelyn Hu

**Figure:**
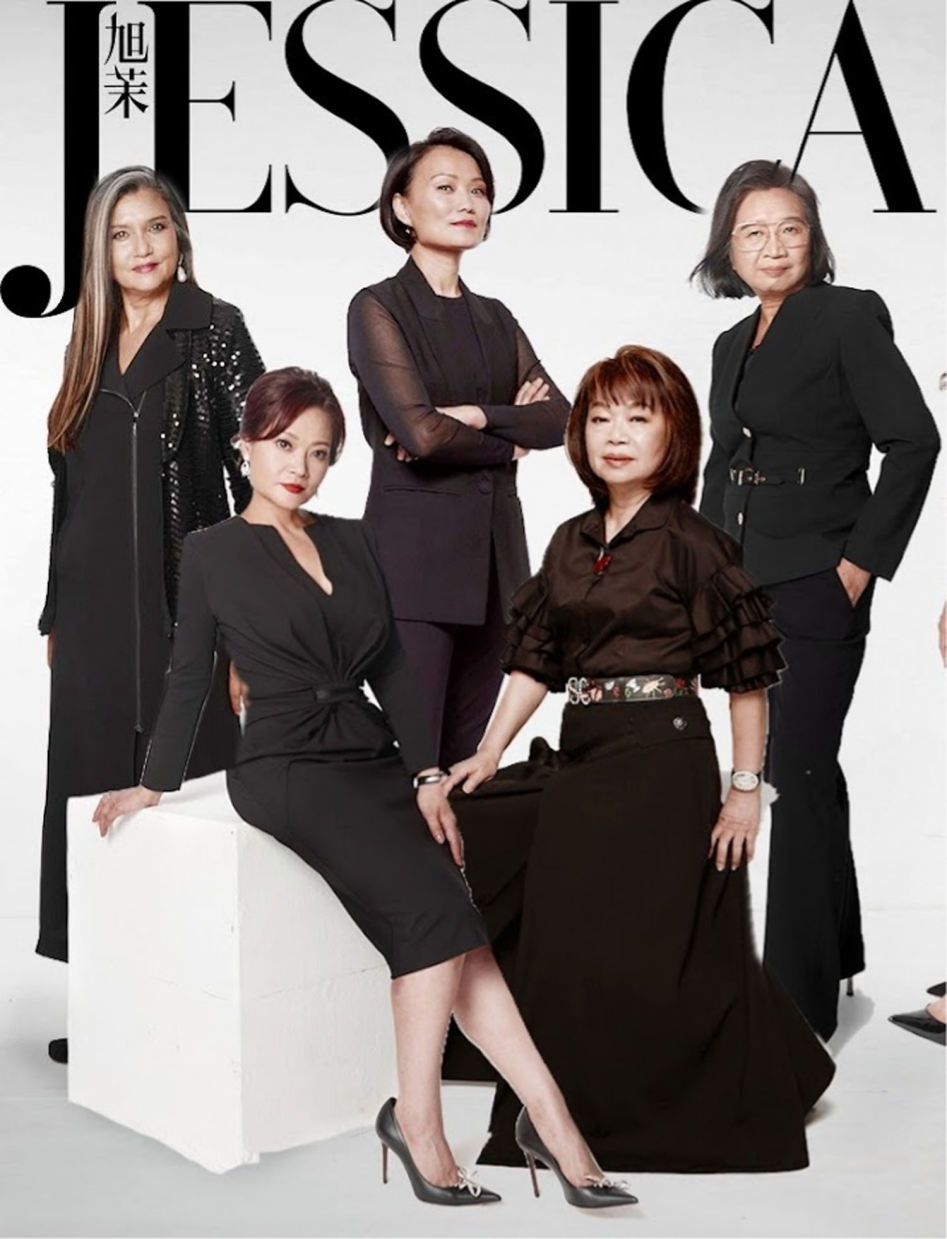
Prof. Lau featured in local magazines in HK

**Q21: In 1993, you became first woman promoted to full professor in the College of Engineering at UMass - a status shared by only about 25 women nationwide at that time. What difficulty have you encountered as a female scientist during those days?**

**A21:** Back then, I focused on doing my best without dwelling on the gender aspect. However, as I saw more women join the faculty, I noticed a pattern: women of my generation often had to achieve more and work longer for the same level of recognition and reward as their male counterparts.

**Q22: The push for greater female representation in science and engineering is a significant movement. What barriers still exist for women in engineering, and how can we better encourage their participation?**

**A22:** Role models are essential for encouraging females to consider engineering. The main hurdle is the stereotype held by many parents about girls not fitting into engineering roles. Here in Hong Kong, girls perform exceptionally well academically, giving them a plethora of career options. Parents often advise them to pursue medicine or business, mistakenly believing that business school is a sure path to wealth. So, fewer girls choose science and engineering, which are seen as tough subjects. Plus, they’re often influenced by their parents’ preferences. To change this, we need to challenge these stereotypes and showcase the successes of women engineers.

**Q23: As you look to the future, what aspirations do you hold for yourself and for the upcoming generation of female scientists?**

**A23:** Identify promising research problems, work on and hopefully solve challenging and impactful research problems, and then work with young people and motivate them.

**Q24: With your experience in editorial roles, what advice can you offer to the journal Light: Science & Applications?**

**A24:** The journal is performing admirably. My suggestion would be to continue the excellent work and maintain the high standards that have been set.

